# COVID-19 vaccine update: vaccine effectiveness, SARS-CoV-2 variants, boosters, adverse effects, and immune correlates of protection

**DOI:** 10.1186/s12929-022-00853-8

**Published:** 2022-10-15

**Authors:** Wei-Yu Chi, Yen-Der Li, Hsin-Che Huang, Timothy En Haw Chan, Sih-Yao Chow, Jun-Han Su, Louise Ferrall, Chien-Fu Hung, T.-C. Wu

**Affiliations:** 1 Physiology, Biophysics and Systems Biology Graduate Program, Weill Cornell Medicine, New York, NY USA; 2 Department of Molecular and Cellular Biology, Harvard University, Cambridge, MA USA; 3 Tri-Institutional PhD Program in Chemical Biology, Memorial Sloan Kettering Cancer Center, New York, NY USA; 4 International Max Planck Research School for Immunobiology, Epigenetics and Metabolism (IMPRS-IEM), Max Planck Institute of Immunobiology and Epigenetics, Freiburg, Germany; 5 Department of Urology, Medical Center, University of Freiburg, Freiburg, Germany; 6 Faculty of Biology, University of Freiburg, Freiburg, Germany; 7 Downstream Process Science, EirGenix Inc., Zhubei, Hsinchu, Taiwan R.O.C.; 8 Department of Pathology, Johns Hopkins University, Baltimore, MD USA; 9 Department of Oncology, Johns Hopkins University, Baltimore, MD USA; 10 Department of Obstetrics and Gynecology, Johns Hopkins University, Baltimore, MD USA; 11 Department of Microbiology and Immunology, Johns Hopkins University, Baltimore, MD USA; 12 The Johns Hopkins Medical Institutions, CRB II Room 309, 1550 Orleans St, MD 21231 Baltimore, USA

**Keywords:** COVID-19, Vaccine, Immunity, Variant, SARS-CoV-2

## Abstract

Coronavirus Disease 2019 (COVID-19) has been the most severe public health challenge in this century. Two years after its emergence, the rapid development and deployment of effective COVID-19 vaccines have successfully controlled this pandemic and greatly reduced the risk of severe illness and death associated with COVID-19. However, due to its ability to rapidly evolve, the SARS-CoV-2 virus may never be eradicated, and there are many important new topics to work on if we need to live with this virus for a long time. To this end, we hope to provide essential knowledge for researchers who work on the improvement of future COVID-19 vaccines. In this review, we provided an up-to-date summary for current COVID-19 vaccines, discussed the biological basis and clinical impact of SARS-CoV-2 variants and subvariants, and analyzed the effectiveness of various vaccine booster regimens against different SARS-CoV-2 strains. Additionally, we reviewed potential mechanisms of vaccine-induced severe adverse events, summarized current studies regarding immune correlates of protection, and finally, discussed the development of next-generation vaccines.

**Supplementary Information:**

The online version contains supplementary material available at 10.1186/s12929-022-00853-8.

## Introduction

Since its first emergence in Wuhan, China in December 2019, Severe Acute Respiratory Syndrome Coronavirus 2 (SARS-CoV-2) and its disease COVID-19 (Coronavirus Disease 2019) have rapidly become a global pandemic and posed a severe threat to public health. By August 2022, SARS-CoV-2 has infected over 580 million individuals and resulted in over 6 million deaths worldwide [1]. In order to control this pandemic, academia, industry, and governments across the world have worked together to make effective vaccines available at an unprecedented speed. The forerunner COVID-19 vaccines—Comirnaty, Spikevax, and Vaxzevria—were granted Emergency Use Authorization (EUA) in December 2020, less than a year from the outbreak. As ccines approved or granof August 2022, there were 40 vated EUA worldwide with over 11 billion doses administered [1–3]. Despite these incredible achievements, new problems have emerged that challenge the long-term control of the pandemic, which include emerging viral variants with increased transmissibility and immune escape; waning immunity over time in vaccinated individuals; and rare but potentially severe vaccine-associated adverse events.

In order to deal with these new challenges, researchers in the scientific and medical fields have conducted numerous follow-up studies on SARS-CoV-2 virus and COVID-19 vaccines which were not covered in our previous review article [4]. We believe that many of these new findings will be fundamental for the improvement of future COVID-19 vaccines; therefore, we hope to summarize this essential knowledge in the current review article. Here, we provided an update on (1) the current landscape of COVID-19 vaccines, (2) the biology of SARS-CoV-2 variants and subvariants, (3) the effectiveness of homologous and heterologous COVID-19 booster vaccinations, (4) the adverse effect of COVID-19 vaccines, (5) current evidence for SARS-CoV-2 immune correlate of protection, and (6) efforts in developing next-generation COVID-19 vaccines.

## Current COVID-19 vaccine landscape

**Fig. 1:**
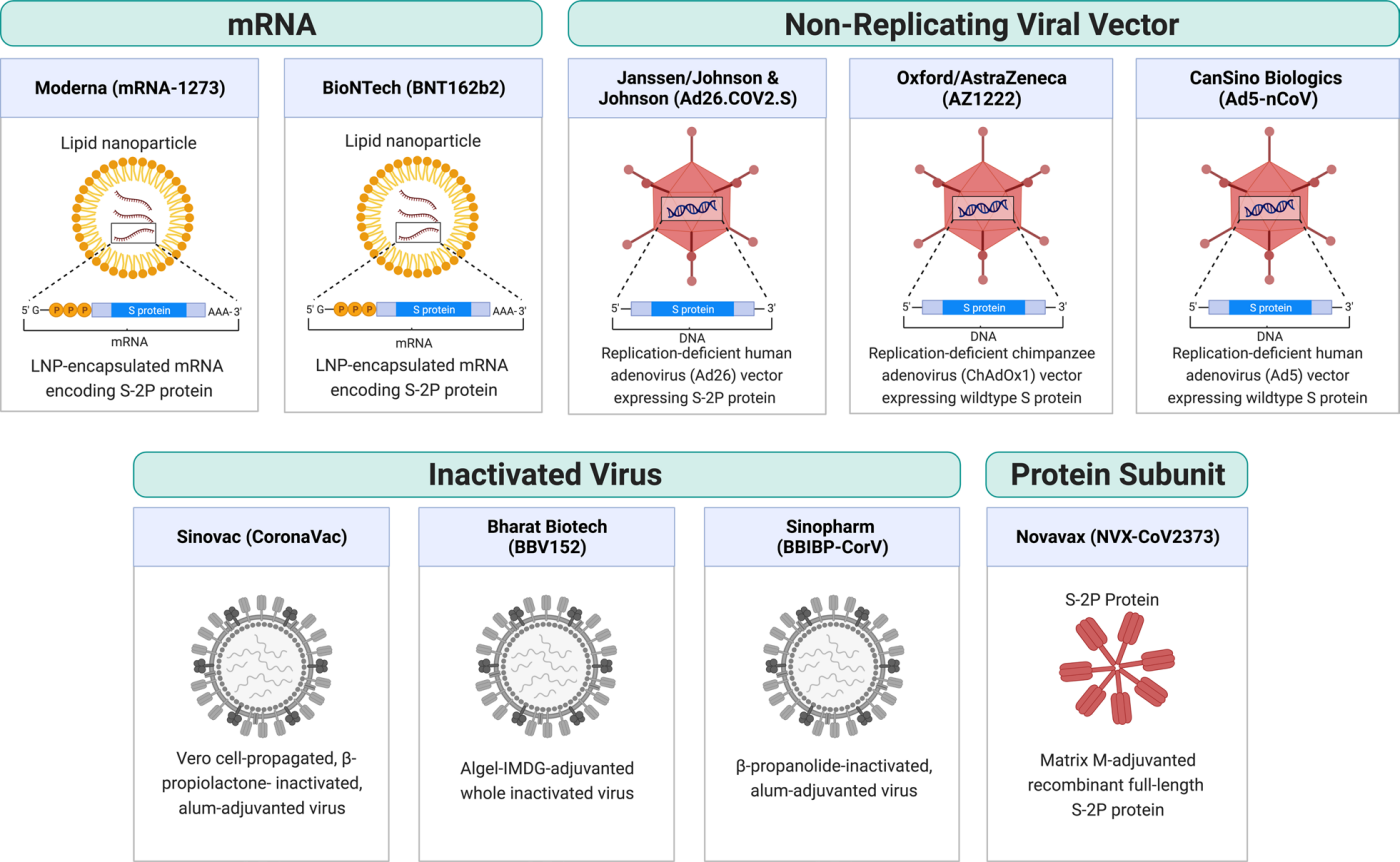
Components of Vaccines with WHO EUL. Covid-19 vaccines with WHO EUL are grouped into four main categories based on the component of individual vaccines: RNA, inactivated virus, non-replicating viral vector and protein subunit. (Created with BioRender.com)

As of August 2022, there are 220 vaccine candidates in phase I to phase III clinical trials and 40 vaccines approved in at least one country worldwide, of which 11 vaccines have been granted Emergency Use Listing (EUL) by the World Health Organization (WHO) [2, 3, 5]. These 11 vaccines include two RNA vaccines (Comirnaty and Spikevax), four viral vector vaccines (Vaxzevria, Covishield, Ad26.COV2.S, and Convidecia), three inactivated virus vaccines (Covilo, CoronaVac, and Covaxin), and two protein subunit vaccines (Nuvaxovid and Covovax) (Table 1). Figure 1 shows a schematic diagram of the different types of vaccine.

**Table 1:** Vaccine list and efficacy (as of August 2022)

	Manufacturer	Vaccine	Platform	No. of Countries in Use	Efficacy* (Infection)	Efficacy* (Severe)	References
1	Moderna	Spikevac (mRNA-1273)	RNA	87	93.2%	98.2%	[19]
2	Pfizer/BioNTech	Comirnaty (BNT162b2)	RNA	146	91.3%	96.7%	[11]
3	Janssen (Johnson & Johnson)	Ad26.COV2.S	Non Replicating Viral Vector	111	52.4%	74.6%	[29]
4	Oxford/AstraZeneca	Vaxzevria (ChAdOx1 nCoV-19, AZD1222)	Non Replicating Viral Vector	141	74.0%	100%	[269]
5	Serum Institute of India	Covishield (Oxford/AstraZeneca formulation)	Non Replicating Viral Vector	49			
6	Bharat Biotech	Covaxin (BBV152)	Inactivated	14	77.8% (symptomatic), 63.6% (asymp)	93.4%	[47]
7	Beijing Institute of Biological Products/Sinopharm	Covilo (BBIBP-CorV)	Inactivated	91	78.1%	100%	[36]
8	Sinovac Biotech	CoronaVac (PiCoVacc)	Inactivated	56	50.7% (Brazil) 65.3% (Indonesia) 83.5% (Turkey)	100% (Brazil)	[39–41]
9	Novavax	Nuvaxovid (NVX-CoV2373)	Protein subunit	38	89.7 (UK) 90.4% (US&Mexico)	100%	[48, 49]
10	Serum Institute of India	COVOVAX (Novavax formulation)	Protein subunit	5			
11	CanSino Biologics	Convidecia (AD5-nCoV)	Non Replicating Viral Vector	10	57.5%	91.7%	[33]

^*^Efficacy represents performance under ideal and controlled trials

In this section, we introduce the components and the trial efficacies of the vaccines. It should be noted that efficacy refers to vaccine performance in controlled trials, whereas effectiveness is drawn from real-world observations. Vaccine efficacies cannot be readily compared because the efficacy can be affected by factors including the vaccine itself (e.g., vaccine type, dose, and number of doses, etc.), the virus (e.g., the virulence of the viral variant at the time of trial), and the participants (e.g., different ages or health conditions).

### RNA vaccine

RNA vaccines consist of viral antigen-encoding messenger RNA (mRNA) encapsulated and stabilized by lipid nanoparticles. Once delivered to cells, they drive transient expression of antigens that are then recognized by the immune system. RNA vaccines have the advantages of ease of design, rapid mass production, and induce both humoral and cellular responses [6]. Therefore, despite being a relatively new technology that had not been previously approved for any use, RNA vaccines were rapidly developed. RNA vaccines for COVID-19 became one of the first authorized and most widely used COVID-19 vaccines.

Comirnaty (tozinameran, BNT162b2) is an RNA vaccine co-developed by BioNTech and Pfizer. The vaccine is a lipid nanoparticle-formulated, N1-methylpseudouridine (m1Ψ)-modified mRNA that encodes full-length transmembrane spike protein with two proline substitutions at residues K986 and V987 to stabilize the protein in perfusion conformation (S-2P) [7–10]. The vaccine efficacies were 91.3% against COVID-19 infection and 96.7% against severe disease through 6 months of follow-up in a large-scale clinical study (NCT04368728) [11]. BNT162b2 is one of the most widely used vaccines around the globe, and it is authorized in 146 countries as of August 2022. Due to the concern over declining protection observed in both Israel and the United States [12, 13], a booster dose was authorized by the US Food and Drug Administration (FDA) based on immunogenicity data demonstrating a 3.3-fold ratio of neutralizing antibody geometric mean titer (GMT) 1 month after the booster dose relative to 1 month after primary series (NCT04368728) [14]. BNT162b2 has also been authorized by the US FDA for use in adolescents and children over 6 months of age at lower doses (10 µg for 5–11 years old and 3 µg for 6 months–4 years, comparing to 30 µg regular dose) based on clinical trials showing over 90% efficacy against infection [15–17].

Spikevax (elasomeran, mRNA-1273) is an RNA vaccine developed by Moderna in partnership with the US National Institutes of Health. It contains lipid nanoparticle-formulated m1Ψ-modified mRNA encoding the S-2P antigen [18]. In clinical trials, the vaccine was reported to be 93.2% effective in preventing COVID-19 illness and 98.2% effective in preventing severe disease [19]. mRNA-1273 has been authorized in at least 87 countries as of August 2022. FDA authorized a half-dose (50 µg) booster shot based on immunogenicity data from a clinical trial (mRNA-1273-P201B, NCT05137236) showing that the neutralizing antibody GMT 1 month after booster dose was 1.8-fold relative to 1 month after primary series [20]. mRNA-1273 was authorized by the US FDA for use in adolescents and children over 6 months of age at lower doses (50 µg for 6–11 years old and 25 µg for 6 months–5 years old) after clinical trials showed more than 90% efficacy against infection [21, 22].

### Viral vector vaccine

Viral vector vaccines employ unrelated, modified viruses as vaccine vectors to deliver antigen-coding genes into the host cells in order to stimulate an immune response. Because the mode of action mimics a natural infection, these vaccines induce potent antibody and T cell responses. Viral vectors have become a versatile platform technology for vaccine development because the viral genome can be easily manipulated to express any antigen of interest.

Vaxzevria (AZD1222, ChAdOx1 nCoV-19, or ChAdOx1) is a vaccine designed by the University of Oxford and produced by AstraZeneca. The vaccine is also produced by the Serum Institute of India under the name Covishield. It is composed of a recombinant, replication-deficient chimpanzee adenovirus (ChAdOx1) vector encoding the full-length wild-type SARS-CoV-2 spike protein with an N-terminal tissue plasminogen activator (tPA) signal sequence that traffics the protein to the cell surface [23]. ChAdOx1 expresses abundant native-like trimeric spike proteins in prefusion conformation on the cell surface [24]. A phase III study reported 74.0% overall efficacy and 100% protection from severe COVID-19 illness [25]. Because ChAdOx1 is cheaper and stable at normal refrigerated temperature, it is one of the most widely distributed COVID-19 vaccines, authorized in 141 countries as of August 2022. Additionally, a phase II study from the UK reported similar tolerance and immunogenicity on ChAdOx1 in children aged 6–17 years compared to adults [26]. Besides, a booster dose was reported to significantly increase neutralizing antibody titer [27] but AstraZeneca did not solicit regulatory authorization for a booster dose of its vaccine. In several countries such as the UK and Australia, mRNA vaccines (BNT162b2 and mRNA-1273) are given as the booster dose for people who were vaccinated with ChAdOx1.

The Ad26.COV2.S vaccine is developed by Johnson & Johnson and Beth Israel Deaconess Medical Center. It consists of a recombinant, replication-deficient human adenovirus type 26 vector encoding full-length spike protein with the wild-type signal sequence, 2P, and furin cleavage site mutations [28]. In a final analysis, the vaccine efficacy was 52.4% against symptomatic COVID-19 and 74.6% against severe–critical disease at least 28 days after administration [29]. An Ad26.COV2.S booster dose given 2 months after the primary dose was found to substantially increase protection; showing 75% efficacy against symptomatic and 100% against severe/critical COVD-19 illness [30]. The US FDA authorized the use of this vaccine as a homologous and heterologous booster before its use was later paused due to concerns over rare blood clotting cases [31]. The Ad26.COV2.S has not been authorized for use in children and adolescents.

Convidecia (Ad5-nCoV) is a vaccine manufactured by CanSino Biologics. It uses a replication-deficient human adenovirus type 5 as a vector to express the wild-type spike protein of SARS-CoV-2 [32]. At single dose, the vaccine efficacy was 57.5% against symptomatic infection and 91.7% against severe disease at least 28 days post vaccination [33]. An orally inhaled, aerosolized formulation of Convidecia is being developed as an easy-to-administer alternative for booster vaccination, as it was found to be more immunogenic as a heterologous booster for individuals who previously received two doses of CoronaVac [34]. Additionally, a phase II study reported similar tolerance and immunogenicity in children aged 6–17 years compared to adults [35].

### Whole inactivated vaccine

Whole inactivated vaccines consist of cultured viral particles that are inactivated by chemicals or radiation. While these vaccines possess the advantage of containing the whole repertoire of antigens of the inactivated pathogen, they typically produce weaker immune responses with little cellular immunity and thus require additional adjuvants. Nonetheless, thanks to the relatively rapid and simple development, whole-inactivated vaccines for SARS-CoV-2 have been widely used.

Covilo (BBIBP-CorV) is an inactivated virus vaccine developed by the Beijing Institute of Biological Products and the Chinese state-owned company Sinopharm. The vaccine consists of β-propanolide-inactivated, aluminum hydroxide (alum)-adjuvanted virus particles. An interim analysis of a multicenter phase III trial reported 78.1% efficacy against symptomatic COVID-19 [36]. A homologous booster was found to increase sVNT GMT by 15-fold relative to the baseline level before the third dose [37]. Additionally, a phase I/II study reported the safety and immunogenicity of Covilo in participants aged 3–17 years. It was then authorized for use in children in China [38].

CoronaVac (PiCoVacc) is a beta-propiolactone inactivated, Vero cell line propagated, alum-adjuvanted whole inactivated vaccine developed by Sinovac Biotech. A phase III clinical trial in Brazil (NCT04456595) showed 50.7% efficacy against symptomatic COVID-19 [39]. Interim analysis of phase III clinical trials in Turkey (NCT04582344) and Indonesia showed 83.5% and 65.3% efficacy against symptomatic infection, respectively [40, 41]. Additionally, a phase I/II study reported safety and immunogenicity of CoronaVac in subjects aged 3–17 years [42]. Some studies found that CoronaVac induces lower antibody responses when compared to mRNA or viral vector vaccines. CoronaVac induces lower levels of nAbs than BNT162b2 (PRNT_50_ GMT of 69.45 versus 251.6) [43] and neutralizing antibody (nAb) levels decline more rapidly than ChAdOx1 [44]. Although a third dose of the same vaccine was found to effectively restore nAb levels [45], such homologous boosting does not provide sufficient levels of protective nAb against Omicron when compared to heterologous boosting with BNT162b2 [46].

Covaxin (BBV152) is a whole inactivated vaccine adjuvanted by alum containing a toll-like receptor 7/8 agonist (Algel-IMDG) developed by Bharat Biotech. An interim analysis of phase III clinical trial in India (NCT04641481) showed 77.8% and 93.4% efficacy against infection and severe disease, respectively [47]. In Feb 2022, a phase III study was launched in the US to test the vaccine as a booster (NCT05258669).

### Protein subunit vaccine

Protein-based vaccines deliver recombinant parts of pathogenic proteins along with an adjuvant to stimulate the immune response. They are relatively safer than inactivated virus vaccines and better tolerated than new mRNA and viral vector technologies. Novavax vaccine (NVX-CoV2373, marketed as Nuvaxovid by Novavax or Covovax by Serum Institute of India) is the first protein-based COVID-19 vaccine that has been authorized for use by the WHO. It contains recombinant 2P and furin cleavage site-mutated full-length spike protein formulated into nanoparticles using a saponin-based adjuvant (Matrix-M). The vaccine efficacy was found to be 89.7% in a British phase III trial and 90.4% in a US and Mexico phase III trial [48, 49]. The latter trial (NCT04611802) has been extended to study a booster dose of the vaccine [50].

## Variants and subvariants of SARS-CoV-2

**Fig. 2:**
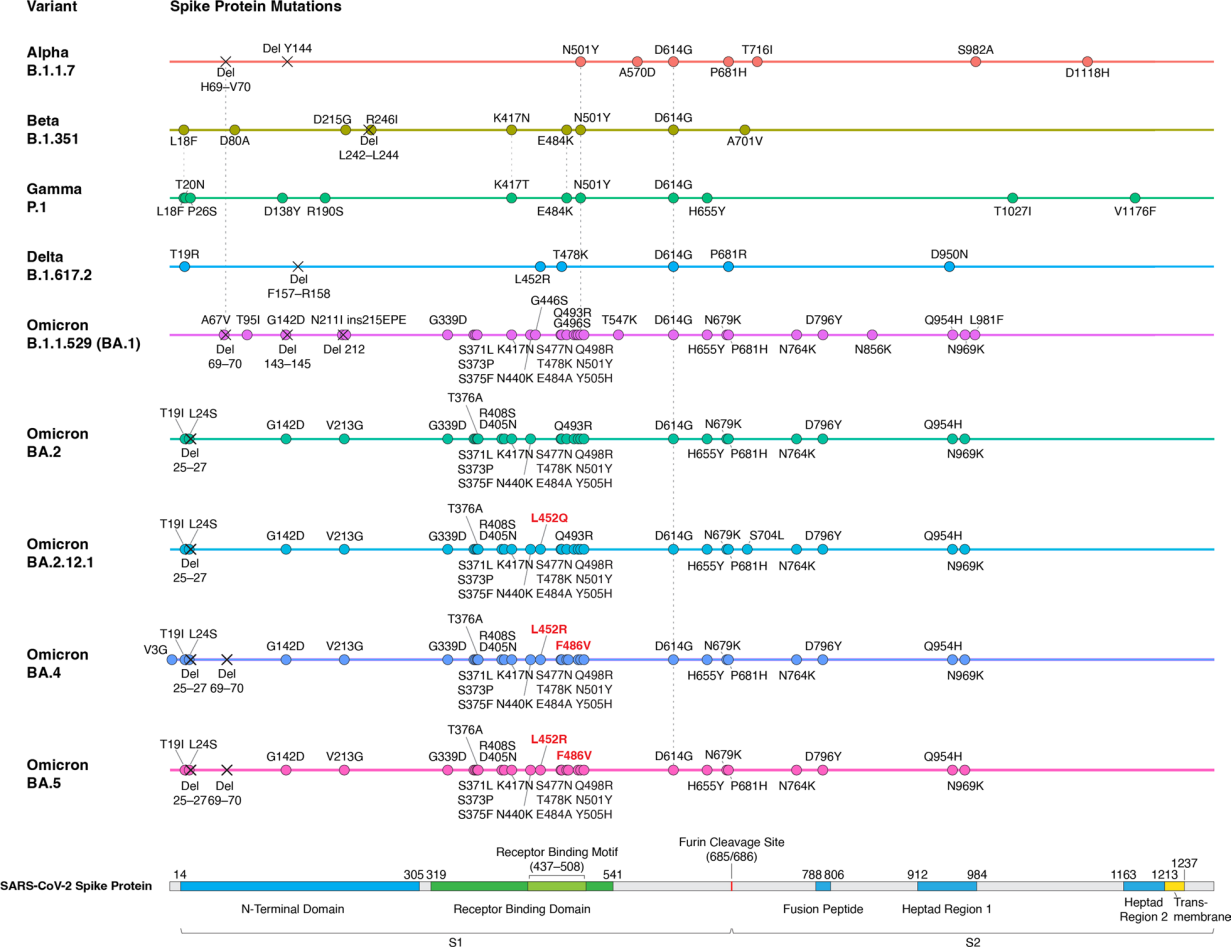
Viral variant and mutations. Amino acid alterations to the spike protein in SARS-CoV-2 VoCs. Domain composition of SARS-CoV-2 spike protein is shown in the bottom [118, 291]. Circles indicate point mutations or insertions; crosses indicate deletions

Since the appearance of SARS-CoV-2 in December 2019, it has evolved more than 10 variant strains [51]. Among these variants, five of them (Alpha, Beta, Gamma, Delta, and Omicron) were thought to be more transmissible and/or more lethal than the original Wuhan strain and have been designated as variants of concern (VoC) by the WHO (Fig. 2) [51]. Some of the variants’ mutations have been shown to be associated with evasion of innate immunity and antibody response, making them more likely to escape from protective effects induced by vaccination or previous infection [52]. In 2022, Omicron has replaced the other variants to become the main circulating variant for most new COVID cases [51]. As a result, the Alpha, Beta and Gamma variants have been removed from the WHO VoC list in March 2022, and the Delta variant has also been removed from the VoC list in June 2022 [51]. For the completeness of this review, we will discuss all the previously designated VoCs and highlight the mechanistic basis of their key mutations.

Alpha, Beta, Gamma, Delta and Omicron variants all contain the D614G mutation, which is a mutation identified early in the pandemic and became prevalent in almost every new SARS-CoV-2 variant, indicating that this mutation provides SARS-CoV-2 some fitness advantages [53–55]. Several cellular and animal studies have shown that the D614G mutation can enhance both the viral replication and infectivity of SARS-CoV-2 [56–58]. Mechanistically, the D614G mutation could reduce the spontaneous S1 shedding from viral particles, making RBD stay in an open conformation, and creating a new protease cleavage site that might increase the spike protein processing. These mutation effects could facilitate viral replication and spike–angiotensin-converting enzyme 2 (ACE2) interaction [56, 59–61]. Although D614G could provide SARS-CoV-2 fitness advantages, there is no evidence that D614G is associated with the severity of COVID-19 disease or the viral escape from the immune system [57, 58].

The first VoC, Alpha (B.1.1.7), was found in the United Kingdom in September 2020 and was designated VoC in December 2020 [62]. In addition to D614G, Alpha has about 10 mutations in the spike protein (Fig. 2), including functionally important N501Y, ΔH69/V70, and P681H mutations [53]. N501Y has been shown to strengthen spike protein binding to receptor ACE2 and thus increase infectivity [63, 64]. ΔH69/V70 mutation has been shown to increase cleaved S2 and spike infectivity [65]. The role of P681H is relatively controversial. Some studies predicted that the mutation could facilitate S1/S2 cleavage due to its adjacent location to the furin cleavage site, while another study provided evidence that P681H did not significantly affect furin cleavage, viral entry, or cell–cell spread [53, 66, 67]. Alpha has been shown to increase transmissibility (as measured by reproduction number) by ~ 30%, increase the risk of hospitalization (median HR: ~ 1.5), severe disease (median HR: ~ 1.65) and mortality (median HR: ~ 1.5–2) [68–71]. Unsurprisingly, it has become the predominant SARS-CoV-2 strain in many countries after its identification in late 2020. The variant then declined in prevalence after the emergence of the more threatening Delta variant in the middle of 2021 [72]. Although the mutations in Alpha contribute to its increased infectivity and virulence, they do not seem to affect the binding of neutralizing antibodies. This is consistent with the observation that the commonly used COVID-19 vaccines, including BNT162b2, mRNA-1273 and ChAdOx1, do not show decreased effectiveness against Alpha-induced symptomatic infection and severe COVID-19 diseases (Table 2; Additional file 1: Table S1) [73–77].

**Table 2:** Vaccine effectiveness against SARS-CoV-2 Variants

Vaccine	Alpha		Beta		Gamma		Beta or Gamma		Delta		Omicron	
	Infection/symptomatic	Severe disease	Infection/symptomatic	Severe disease	Infection/symptomatic	Severe disease	Infection/symptomatic	Severe disease	Infection/symptomatic	Severe disease	Infection/symptomatic	Severe disease
AstraZeneca ChAdOx1 nCoV-19	70.4–87 [73, 76, 77]	82–86 [77, 270]	10.4^‡^ [107]						60–82.8 [76, 77, 93, 128, 235, 271]	90–95.2 [77, 128]	48.9 [93]	
Pfizer-BNT BNT162b2	76–95.3 [76, 77, 129, 220, 270, 272–275]	85–100 [77, 129, 270, 272]	86–100 [11, 77, 129]	92–100 [77, 129]	90 [77]	94 [77]	89 [77]	95 [77]	42–93 [76, 77, 93, 128, 235, 272, 276–278]	75–98.7 [77, 128, 272, 278]	55.2–70 [93, 94, 279]	
Moderna mRNA-1273	86–100 [75, 77, 272, 275, 280]	91.6–95 [77, 272]	96.4 [280]		95.5 [75]		88 [77]		73.1–94 [75, 77, 93, 127, 272, 278]	81–99 [77, 127, 272, 278, 281]	36.7–94.5 [279]	84.5 [281]
Johnson & Johnson Ad26.COV2.S	70.2* [29]		51.9*–64* [29, 282]	81.7* [29, 282]	36.5*–68.1* [29, 282]	87.6* [282]			69.9^†^ [127]	93.7^†^ [127]		
Novavax NVX-CoV2373	85.6–86.3 [48, 283]		51–60 [283, 284]						82 [285]			
Sinovac Biotech CoronaVac					37.1^‡^ [286]							
Any mRNA (BNT162b2 or mRNA-1273)	86–90 [74, 287]	94 [287]					77–88 [74, 287]	100 [287]	66–92 [280, 288, 289]	94 [288, 289]	36–69 [288, 289]	55–71 [288, 289]
CoronaVac or BBIBP-CorV									59 [290]			
BBV152									65.2* [47]			

Vaccine		Delta		Omicron	
Primary	Booster	Infection/symptomatic	Severe disease	Infection/symptomatic	Severe disease
ChAdOx1 nCoV-19	BNT162b2	94 [93]		71 [93]	
BNT162b2	BNT162b2	88–93 [93, 177]	97 [177]	76 [93]	89 [176]
Any mRNA	Any mRNA	93 [139]	94 [178]	67 [139]	90 [178]
mRNA-1273	mRNA-1273	94 [281]	> 99 [281]	72 [281]	> 99 [281]

^*^Efficacy in trial

^†^Age 18–49 yr

^‡^Negative 95% CI lower bound value

Summary of Effectiveness of Vaccine Booster Against SARS-CoV-2 Variants

The next two VoCs, Beta (B.1.351) and Gamma (P.1), were identified in 2020 in South Africa and Brazil, respectively [62]. They were designated as VoC almost simultaneously in January 2021 [62]. Beta and Gamma have been shown to have increased transmissibility by ~ 25% and ~ 38%, respectively [68]. In addition, Beta has a significantly increased risk of hospitalization (median HR: ~ 2.2), ICU admissions (median HR: ~ 2.2) and mortality (median HR: ~ 1.5); Gamma also has an increased risk of ICU admissions (median HR ~ 1.95) [78]. These two VoCs also contain the same D614G and N501Y mutations as Alpha. They also both have E484K mutation (E484K is also found in some sub-lineages of Alpha), and contain mutations on lysine 417 (K417N for Beta and K417T for Gamma) (Fig. 2; Additional file 1: Table S1) [62]. The E484K mutation has been predicted to destabilize the native conformation of the RBD tip and has been shown to help ​​SARS-CoV-2 evade neutralizing antibodies [79–81]. The K417 of spike protein can form salt-bridge interaction with various antibodies, and mutating this residue would cause disrupted RBD-antibody interaction that could lead to viral escape from human antibodies [81, 82]. Even though these mutations have been demonstrated to increase viral escape from antibodies in vitro, Beta and Gamma only show a mild decrease in vaccine effectiveness in preventing symptomatic infection and almost no effect to the vaccine effectiveness of severe disease protection (Table 2; Additional file 1: Table S1) [74, 77].

The Delta (B.1.617.2) variant was first discovered in India in October 2020 and was designated as VoC in May 2021 [51]. Compared with previously identified VoCs, Delta was significantly more transmissible (reproduction number increased by ~ 100%) and associated with a higher risk of hospitalization (median HR ~ 2.1), admission to ICU (median HR ~ 3.35) and mortality (median HR ~ 2.3) [68, 78]. Due to its higher risk associated with severe disease and mortality, Delta became more problematic and gained more attention than Alpha, Beta and Gamma. After its appearance, Delta soon substituted Alpha to become the prevailing strain worldwide [83]. Delta does not have N501Y or K417T/N mutations that are found in previously identified variants. However, it contains many other mutations in the spike protein, including L452R, T478K, and P681R (Fig. 2) [83]. The L452R mutation has been shown to promote viral replication and increase spike stability, viral infectivity, and viral fusogenicity [84, 85]. The T478 residue is located in the receptor-binding motif, and its mutation to lysine might lead to the enhanced binding affinity of RBD to ACE2 [83, 86]. The P681R mutation has been shown to facilitate cleavage of the spike protein, enhance viral fusogenicity, and lead to higher pathogenicity [83, 87]. All these newly occurring mutations could contribute to the increased transmissibility and higher risk of severe COVID-19 disease. Vaccine effectiveness studies have shown that one dose of either BNT162b2 or ChAdOx1 has lower effectiveness against Delta compared to Alpha [75, 76]. However, after the two-dose vaccinations, the effectiveness is much higher and closer to the results against Alpha (Table 2; Additional file 1: Table S1) [75–77]. Additionally, studies focusing on adolescents between 12 and 17 years of age showed that two doses of BNT162b2 have similar effectiveness and were highly effective against Covid-19–related hospitalization and ICU admission caused by Delta (Additional file 1: Table S2) [88, 89]. Therefore, the recommended two-dose mRNA and viral vector vaccines are still effective in protecting against symptomatic infection and severe diseases caused by Delta.

Finally, the Omicron (B.1.1.529) variant was identified in South Africa in November 2021 and was designated as VoC in the same month due to its sharp increase in reported infections (Fig. 2) [90]. As of August 2022, Omicron has already been identified in 190 countries and has rapidly outpaced Delta in driving the upsurge of COVID cases in most countries [91]. Since its discovery, the Omicron variant has continued to evolve from its original BA.1 strain, leading to multiple descendent lineages. Some of these Omicron subvariants have shown to be more transmissible than the others, prompting WHO to add a new category to its variant tracking system, named “Omicron subvariants under monitoring" to let public health authorities know which VoC lineages need further attention [51]. There have been seven lineages designated as Omicron subvariants under monitoring, including BA.2.12.1, BA.2.9.1, BA.2.11, BA.2.13, BA.2.75, BA.4, and BA.5. The first five belong to BA.2 sublineage, and the last two are BA.1 and BA.2 sister lineages.

**Fig. 3:**
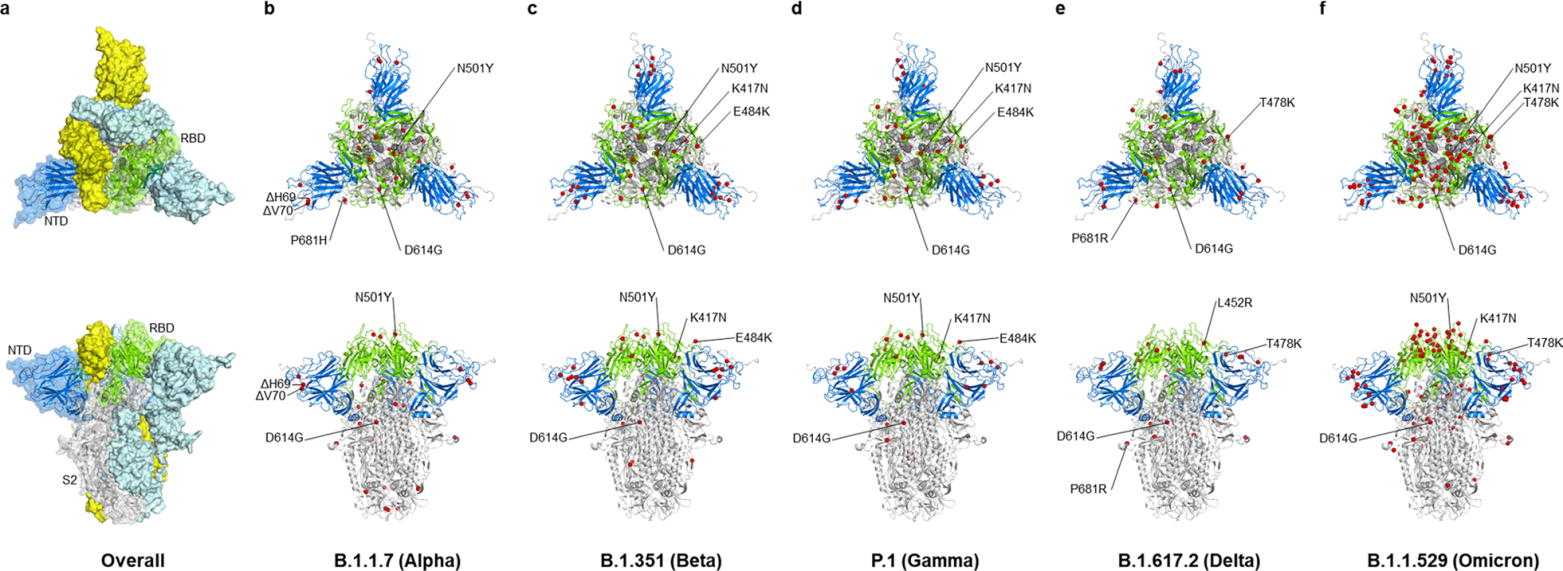
The cartoon depiction of the SARS-CoV-2 spike protein trimer and the mutating amino acids. The 3D structure of SARS-CoV-2 spike protein trimer in the closed prefusion configuration (modified from PDB 6VXX) is shown in top (left panel) and side views (right panel). **a** One of the spike protein monomers is shown in the ribbon diagram with the NTD, the RBD, and the S2 domain of the spike protein are colored in blue, green and gray, respectively. Whereas the other two spike monomers are shown as surface and colored in cyan and yellow, respectively. **b**–**f** The mutating amino acid residues in each variant are highlighted as red spheres. The NTD, RBD, and the S2 domain of the spike protein are colored in blue, green and grey in the ribbon diagram, respectively

The reason why Omicron has quickly substitute Delta as the prevailing VoC can be partially attributed to its capability to escape from infection- and vaccine-induced neutralizing antibodies [92]. One study has shown that vaccine effectiveness against Omicron-induced symptomatic disease dropped to less than 20% (while the effectiveness remains 40–80% for Delta) at 25 weeks after two doses of ChAdOx1, BNT162b2, or mRNA-1273 vaccination [93]. Another South African study also showed that the protective effect of two-dose BNT162b2 against symptomatic disease dropped from 90 to 70% during the peak Omicron period [94]. Additionally, multiple observational studies focusing on children and adolescents after the omicron variant (B.1.1.529) emerged also showed a lower protective effect of BNT162b2 [95–99]. Despite the protection waning, it still protects against multisystem inflammatory syndrome (MIC) in children, which is a rare COVID infection causing hyperinflammation among persons aged 12–18 years (Additional file 1: Table S2) [100]. Omicron has more than 30 mutations in the spike proteins, which contains 2–3 times more mutations than Alpha and Delta (Figs. 2 and 3) [92]. These mutations are mostly sitting in two domains targeted by neutralizing antibodies: the receptor-binding domain (RBD) and the N-terminal domain (NTD), which explains why Omicron is more effective in escaping vaccine-induced immunity [101–104]. Another reason why Omicron is rapidly spreading could be explained by its different modes of infection [92]. Scientists have found that, unlike most SARS-CoV-2 variants that are dependent on TMPRSS2 for infecting cells, Omicron tends to efficiently use a TMPRSS2-independent endosomal route of entry, which makes it more capable of infecting many low TMPRSS2-expressing cells in the upper respiratory tract [105]. If Omicron lingers in the upper airways, viral particles are more easily spread by material expelled from the nose and mouth, allowing it to transmit at a faster pace [105]. This TMPRSS2-independent infection mode could also contribute to the clinical finding that Omicron is less likely to cause severe disease compared with other VoCs, as Omicron preferentially infects and replicates in the airway above the lungs and is not infecting TMPRSS2-rich lung cells as strongly as other variants [106–111]. Notably, another study provided evidence that Omicron viruses are less effective at antagonizing the host cell interferon response than Delta, which provides an alternative explanation for why it causes less severe disease [112].

Since the appearance of the Omicron variant in late 2021, a succession of Omicron subvariants have come and gone. In the U.S, BA.1 strain has been the major subvariant before being replaced by BA.2 in March 2022 [113]. Then BA.2 was also soon substituted by BA.2.12.1 in May 2022 as the dominant subvariant [113]. In July 2022, the next subvariant BA.5 took the lead as the most common subvariant for new COVID cases [113]. There are multiple reasons why new subvariants keep substituting the older ones. For the BA.2 case, several studies compared the antibody neutralization profile between BA.1 and BA.2 and found that BA.2 is only slightly better than BA.1 in immunological escape. This suggests that additional viral characteristics, such as enhanced transmissibility, should also play a role in BA.2’s ascent [103, 114–117]. The BA.2 strain contains 8 unique spike mutations and lacks 13 spike alterations found in BA.1; therefore, scientists haven’t been able to pinpoint the specific genetic mutations responsible for its growth advantage [118]. For the case of BA.2.12.1, BA.4, and BA.5, their selective advantages mainly come from novel mutations in the spike protein that help them evade neutralizing antibodies [119, 120]. BA.2.12.1 possesses a critical L452Q mutation while BA.4 and BA.5 contains both L452R and F486V mutations (Fig. 2) [118]. These mutations have been demonstrated to facilitate escape from neutralizing antibodies induced by vaccination or infection of the older variants [121–124]. Notably, these newly emerged sublineages also have the ability to escape from BA.1 specific antibodies, so infection with earlier Omicron BA.1 strain doesn’t seem to offer effective protection against the BA.2.12.1/BA.4/BA.5 subvariants [121, 123].

In summary, although Omicron and its newly emerged subvariants are more capable of escaping vaccine- or infection-induced immune responses, the risk of Omicron-induced severe disease and mortality is lower than the other variants, making it more similar to seasonal flu than it was at the beginning of the pandemic. The immune escape property of Omicron subvariants also explains why in certain countries such as the U.S. and Europe, they have high vaccine rate but the infection rate is still high. It has been reported that additional booster vaccines and Omicron-specific vaccines are helpful in mitigating the immune evasion problem caused by Omicron and its subvariants, which will be discussed in detail in the following sections [93].

## Vaccine boosters and effectiveness

A booster dose represents a dose of vaccine after the primary vaccination series, i.e., any dose after one dose of Ad26.COV2.S or two doses of ChAdOx1, BNT162b2, or mRNA-1273. In this section, we review the immunogenicity and effectiveness of boosters against viral variants and comparisons of homologous and heterologous boosters (Additional file 3: Table S3).

### Booster dose after complete vaccination

Recent studies have shown that emerging variants such as Delta or Omicron exhibit strong immune evasion to neutralizing antibodies induced by primary vaccination or previous infections [125, 126] and that protective effects wane over time [127, 128]. This declining protection leads to more breakthrough infections in fully vaccinated people [129]. For example, the effectiveness in preventing disease decreased from an initial 90% to 60% in the follow-up studies after 6 months [11]. Therefore, the durability of vaccine-induced protection and the need for periodic booster immunizations have become the center of discussion.

Booster vaccinations offer improved protection against COVID-19 illness. A phase III trial (NCT04955626) showed a third dose of the BNT162b2 vaccine provided 95.3% relative efficacy against COVID-19 compared to two doses of the BNT162b2 vaccine [130]. Many countries started to offer COVID-19 boosters in the last quarter of 2021 when Delta became the predominant variant [131, 132]**.** While boosters effectively provided protection against severe illness and hospitalization caused by Delta, Omicron posed another challenge as the breakthrough infections increased drastically at the end of 2021. Fortunately, boosters were shown to increase serum anti-spike antibody levels and the neutralization titers against Omicron in recipients boosted with BNT162b2 [133–135], mRNA-1273 [136–138], or general mRNA vaccines [139]. In addition, CoronaVac [140] and mRNA vaccine boosters [141] were both shown to increase anti-receptor binding domain-specific memory B cells and rapidly produce antibodies targeting diverse variants such as Omicron in boosted individuals. Although breakthrough infections of Delta and Omicron can still occur in boosted individuals, the viral loads seem to be lower and the symptoms are milder [142, 143].

Booster vaccinations are especially important for vulnerable and high-risk groups. For example, studies showed cancer patients under treatment [144, 145], solid-organ transplant recipients [146], and the elderly [147] need additional BNT162b2 or mRNA-1273 boosters to achieve sufficient protection against Delta and Omicron [148, 149]. In January 2022, Israel started to offer a second booster to the elderly and immunocompromised individuals [150, 151]. The US also started to offer second mRNA boosters to the elderly and people with underlying medical conditions in March 2022 [152, 153]. A retrospective study in Israel showed that the second BNT162b2 booster reduced COVID-19 related hospitalizations and deaths in people who were over 60 years old compared with one booster dose [154].

### Heterologous primary series (mix primary vaccination)

In multiple-dose vaccine series, recipients are usually given the exact same vaccines for each dose. Alternatively, mixing different vaccines with the same antigens in a multiple-dose regimen is known as heterologous primary series (or heterologous prime-boost) vaccination. In the case of COVID-19, this refers to a different vaccine for the second dose in the two-dose (primary series) regimen. Previous studies have shown that heterologous vaccinations can be more immunogenic and provide better protections [155]. However, it was originally unknown whether heterologous vaccinations provide better protection against SARS-CoV-2.

The issue of heterologous vaccinations arose due to the suspension and the shortage of vaccines in some countries. Several European countries halted the use of ChAdOx1 because of concerns about potential blood clotting. Consequently, individuals vaccinated with one dose of ChAdOx1 are offered an mRNA vaccine for their second dose. In addition, heterologous vaccinations provide flexibility in times and areas of limited vaccine supply. Therefore, heterologous COVID-19 vaccination was studied to aid decisions in vaccination campaigns.

Heterologous vaccination was shown to be safe and immunogenic. Multiple studies in adults showed heterologous prime ChAdOx1 dose followed by an mRNA vaccine (either BNT162b2 or mRNA-1273) results in comparable reactogenicity and more robust humoral immunity compared to homologous ChAdOx1 vaccinations [156–160]. A comprehensive phase II trial (Com-COV2) compared the serum profiles of individuals primed with ChAdOx1 or BNT162b2 followed by a second dose of a homologous vaccine, mRNA-1273, or NVX-CoV2373. It showed that heterologous vaccinations generally induced more anti-spike IgG than homologous dosing. Heterologous dosing with mRNA-1273 induced higher anti-spike IgG antibody responses than homologous vaccination in individuals who received first-dose ChAdOx1 or BNT162b2. However, heterologous NVX-CoV2373 dosing only showed non-inferiority in first-dose recipients of ChAdOx1 but not BNT162b2 when compared with homologous vaccinations [161]. Similarly, a French study showed that heterologous BNT162b2 and ChAdOx1 combination offers higher protection than homologous BNT162b2 primary vaccinations in a real-world observational study of healthcare workers [162]. Moreover, a heterologous ChAdOx1 dose after CoronaVac priming was found to yield an anti-spike RBD antibody level that is eightfold higher than homologous CoronaVac and comparable to homologous ChAdOx1 vaccination [163]. Therefore, heterologous vaccination generally provides better protection.

From a mechanistic point of view, a study found that second dosing with mRNA-1273 augmented the ChAdOx1-primed responses onto epitopes in the prefusion-conformation spike protein, resulting in higher neutralizing antibody titers and better protection than homologous ChAdOx1 vaccination [164]. The aforementioned French study also found that ChAdOx1 induced a stronger T cell response but weaker humoral response than BNT162b, suggesting that the two vaccines could complement each other when used in combination [162]. In sum, these studies provide mechanistic insights in favor of heterologous vaccinations.

### Heterologous booster vaccination (additional dose after primary vaccination)

When booster vaccinations were first offered, there was a discussion about whether to receive a homologous or heterologous booster. To assist in the development of better booster strategies, many studies are being performed to identify the immunogenicity and safety of heterologous boosters in fully vaccinated individuals (Table 3, Additional file 4: Table S4).

**Table 3:** Selected heterologous booster studies on mix and match approach

A							
COV-BOOST Trial—Munro et al., 2021 (UK) [165]							
Primary doses	N = 2557		anti-spike IgG, ELU/mL	Pseudovirus neutralizing antibody (GMT) Delta NT50	Live virus neutralizing antibody NT80	Cellular response WT	Cellular response, Delta
ChAd/ChAd	–	93	801	20.0	146	48.1	38.1
ChAd/ChAd	ChAd	100	2457	48.9	346	53.0	44.9
ChAd/ChAd	NVX	96	6975	124	837	113.7	117.9
ChAd/ChAd	NVX (half)	97	4634	87.2	713	98.4	86.3
ChAd/ChAd	–	93	763	20.4	174	42.6	42.2
ChAd/ChAd	BNT	95	20,517	315	4899	115.5	123.2
ChAd/ChAd	VLA	95	1835	35.2	354	52.2	123.2
ChAd/ChAd	VLA (half)	107	1430	31.1	301	55.5	54.7
ChAd/ChAd	Ad26	101	5517	125	1053	106.0	102.1
ChAd/ChAd	–	102	852	18.6	152	39.5	35.2
ChAd/ChAd	BNT (half)	105	16,045	321.3	2501	135.9	139.1
ChAd/ChAd	mRNA	98	31,111	559.7	5421	148.9	152.1
ChAd/ChAd	CVnCoV	105	3996	64.5	774	47.8	45.5
BNT/BNT	-	111	2541	37.9	531	34.5	35.7
BNT/BNT	ChAd	98	13,424	260	2614	95.8	108.0
BNT/BNT	NVX	103	10,862	165	1454	56.6	56.9
BNT/BNT	NVX	99	8550	131	1792	35.3	41.6
BNT/BNT	–	97	3197	56.5	756	29.4	28.2
BNT/BNT	BNT	96	27,242	392	4603	83.8	82.1
BNT/BNT	VLA	99	4204	67.1	836	33.5	29.6
BNT/BNT	VLA (half)	98	3721	54.7	555	38.1	39.2
BNT/BNT	Ad26	89	17,079	418	3535	111.0	121.5
BNT/BNT	–	100	3029	41.6	469	22.0	25.9
BNT/BNT	BNT (half)	94	23,082	352.6	3263	78.4	93.0
BNT/BNT	mRNA	92	33,768	508.7	5354	112.0	118.3
BNT/BNT	CVnCoV	94	7613	119.1	1960	46.7	52.2

B						
**MixNMatch Trial—Atmar et al., 2022 (US)** [167]						
**Primary doses**	**N = 458**		**IgG serum binding antibody titer (GMT)**	**Th1 CD4 + T cell, Median %**	**Th2 CD4 + T cell, Median %**	**Th1 CD8 + T cell, Median %**
BNT/BNT	BNT	50	3164	0.11 > > 0.21	0.00 > > 0.02	0.03 > > 0.11
BNT/BNT	mRNA	51	5231	0.14 > > 0.32	0.00 > > 0.01	0.02 > > 0.08
BNT/BNT	Ad26	53	2600	0.11 > > 0.18	0.00 > > 0.00	0.06 > > 0.13
mRNA/mRNA	BNT	50	5273	0.26 > > 0.33	0.01 > > 0.03	0.02 > > 0.08
mRNA/mRNA	mRNA	51	6224	0.24 > > 0.48	0.00 > > 0.03	0.02 > > 0.06
mRNA/mRNA	Ad26	53	4560	0.34 > > 0.39	0.03 > > 0.02	0.03 > > 0.11
Ad26	BNT	51	2277	0.06 > > 0.21	0.00 > > 0.01	0.17 > > 0.83
Ad26	mRNA	49	2986	0.13 > > 0.26	0.00 > > 0.00	0.32 > > 0.66
Ad26	Ad26	50	369	0.10 > > 0.09	0.00 > > 0.00	0.19 > > 0.15
			* Day 29	* Day 1 > > Day 15 after booster		

C				
Costa Clemens et al., 2022 (Brazil) [166]				
Primary doses	N = 1240		Anti-spike igG	Pseudovirus neutralisation titres (GMR)
CoronaVac/CoronaVac	Ad26	294	6.7	8.7
CoronaVac/CoronaVac	BNT	333	13.4	21.5
CoronaVac/CoronaVac	ChAd	296	7.0	10.6
CoronaVac/CoronaVac	CoronaVac	281	1 (ref)	1 (ref)

BNT: BNT162b2; mRNA: mRNA-1273; ChAdOx1: ChAd; Ad26: Ad26.COV2.S; NVX: NVX-CoV2373; VLA: VLA2001; –: control (3 control groups in total in the trial)

* Calculate as spots per 1 M PBMC

Accumulating evidence has shown that heterologous boosters are generally more immunogenic than homologous ones. Several large trials aimed to compare multiple primary and booster combinations. For example, the phase II COV-BOOST trial in the UK (ISRCTN 73765130) compared different boosters (ChAdOx1, BNT162b2, mRNA-1273, NVX-CoV2373, VLA2001, and CVnCov) in individuals primed with ChAdOx1 or BNT162b2 vaccines. The trial result showed boosted antibody and neutralizing responses in all groups except for VLA2001 after two doses of BNT162b2, and the effect seems to be the best in the group receiving mRNA boosters [165]. A phase IV study in Brazil compared four boosters (ChAdOx1, BNT162b2, Ad26.COV2.S, or CoronaVac) in recipients of two primary doses of CoronaVac and concluded that heterologous boosters induced high concentrations of neutralizing antibodies in pseudovirus assays and virus neutralization assays using the Delta and Omicron variants [166] (Table 3). Another phase I/II MixNMatch trial in the US (NCT04889209) compared three boosters (BNT162b2, mRNA-1273, or Ad26.COV2.S) in individuals fully vaccinated with BNT162b2, mRNA-1273, or Ad26.COV2.S, making a total of nine combinations [167]. Initial results showed all combinations of homologous and heterologous boosters had an acceptable safety profile, increased antibody neutralizing titers and binding titers against SARS-CoV-2 pseudovirus in adults. Importantly, heterologous boosters induced higher titers compared to homologous boosters. Spike-specific T-cell responses increased in all except the homologous Ad26.COV2.S-boosted subgroup. CD8+ T-cell levels were more durable in the Ad26.COV2.S-prime recipients, and heterologous boosting with the Ad26.COV2.S vaccine substantially increased spike-specific CD8+ T cells in the mRNA vaccine recipients [167] (Table 3).

Several studies also compared the immunogenicity of homologous and heterologous boosters in a smaller setting with fewer groups (Additional file 4: Table S4). For example, studies in Hong Kong [46, 168], Thailand [169], and Turkey [170] compared CoronaVac or BNT162b2 boosters after two doses of CoronaVac. Similar to the result of the phase IV study in Brazil [166], all studies show BNT162b2 booster groups have higher levels of SARS-CoV-2 neutralizing, spike binding antibody, and cellular response compared with homologous CoronaVac boosters. Additionally, a phase IV trial in China (NCT04892459) compared CoronaVac and an adenoviral vector vaccine Convidecia (AD5-nCOV, Cansino) following two doses of CoronaVac [171]. Their results also support that heterologous boosting with Convidecia is safe and more immunogenic than homologous boosting. Besides, two observational studies in Chile [172] and Malaysia [173] compared the real-world effectiveness of CoronaVac, ChAdOx1 and BNT162b2 vaccine boosters in individuals who had a primary immunization with CoronaVac or ChAdOx1. Both studies confirmed a higher vaccine effectiveness of heterologous boosting. Furthermore, a trial in the Netherlands (NCT04927936) [174] and an observational study in Singapore [175] compared the homologous or heterologous mRNA boosters in individuals primed with one dose of the Ad26.COV2.S vaccine. Both studies provide evidence that Ad26.COV2.S and mRNA boosters are safe, well tolerated, and provide stronger immune responses, especially after boosting with mRNA-based vaccines.

In sum, most evidence available for WHO-approved vaccines indicates that a heterologous booster is safe, has better immune responses and higher effectiveness than a homologous booster. A heterologous booster vaccination strategy may enhance the overall protection against variants and provide a longer duration of protection.

### Effectiveness of booster vaccination against viral variants

The emergence of more transmissible Delta and Omicron variants has underscored the importance of booster vaccinations, which were shown to increase neutralizing antibodies and immunogenicity against Delta and Omicron in multiple studies. To date, the most comprehensive real-world observations are made in the UK and the US (Table 2, Additional file 3: Table S3). For individuals who received a homologous BNT162b2 booster, the effectiveness against infection with Delta and Omicron was estimated to be 88–93% and 76%, respectively, while the effectiveness against severe disease caused by Delta and Omicron was estimated to be 97% and 89%, respectively [93, 176, 177]. In line with this, the Centers for Disease Control and Prevention (CDC) reports showed that homologous mRNA vaccine (BNT162b2 or mRNA-1273) boosters are highly effective, with effectiveness against infection with Delta and Omicron estimated to be 93% and 67%, respectively; and effectiveness against hospitalizations caused by Delta and Omicron were estimated to be 94% and 90%, respectively [139, 178]. Moreover, in ChAdOx1-primed BNT162b2-boosted individuals, the effectiveness against infection with Delta and Omicron was 94% and 71%, respectively [93]. Observational studies from Israel also showed that BNT162b2 booster was associated with a lower risk of SARS-CoV-2 infection for health care workers, the elderly, and young individuals [134, 179–181]. Therefore, booster doses were found to be highly effective in preventing infection and severe diseases caused by Delta and Omicron variants.

## Adverse effects

Same as common flu vaccines and other vaccines, the most common adverse effects of COVID-19 vaccinations are injection site pain or tenderness, fatigue, headache, rash, fever, chill, muscle pain, and joint pain. These adverse effects are generally mild and self-limiting and are not major concerns. However, there were also severe adverse events (SAEs) reported to be followed by COVID-19 vaccination, including thrombosis and thrombocytopenia, myocarditis or pericarditis, inflammatory myositis, autoimmune diseases such as Guillain-Barré syndrome, and life-threatening allergic reactions known as anaphylaxis [182, 183]. Anaphylaxis can occur after any kind of vaccination and thus is not specific to COVID-19 vaccines. Guillain-Barré syndrome is a rare neurological disorder and is associated with bacterial infection, viral infections, and influenza vaccination. It has also been reported to be higher in recipients of Ad26.COV2.S but not in other vaccines [184]. In this section, we will focus on two severe adverse events including vaccine-induced immune thrombotic thrombocytopenia and myocarditis/pericarditis.

Since many of the COVID-19 vaccines were first used after EUA and there are not any prior approved vaccines using the mRNA platform, it is necessary to monitor any adverse effects reported following the COVID-19 massive vaccination programs. Several surveillance systems offer rapid reports of potential adverse effects, including the Vaccine Adverse Event Reporting System (VAERS), Centers for Disease Control and Prevention Vaccine Safety Datalink, the v-safe app, and the WHO AEFI tool [185, 186]**.** To track all possible adverse events, with or without causal relationships with vaccination, researchers use a neutral term called adverse event following immunization (AEFI), defined as “any untoward medical occurrence which follows immunization and which does not necessarily have a causal relationship with the usage of the vaccine” [187]. Alternatively, adverse events of special interest (AESI) are prespecified adverse events that have the potential to be causally related to COVID-19 vaccines and are monitored more carefully [188].

### Vaccine-induced immune thrombotic thrombocytopenia in adenoviral vector vaccines

Followed by injection of adenoviral vaccines including Oxford–AstraZeneca’s ChAdOx1 and Janssen’s Ad26.COV2.S, there have been reports of rare SAEs such as thrombosis and thrombocytopenia syndrome [189]. These events have been called vaccine-induced immune thrombotic thrombocytopenia (VITT) and are characterized by an atypical presentation of thrombotic events such as cerebral venous sinus thrombosis and splanchnic vein thrombosis [190]. The frequency of VITT associated with the first dose of ChAdOx1 was estimated to be 8 to 38 cases per million doses [191].

There are several possible mechanisms of VITT. One possible explanation is that the local inflammatory mediators such as vasodilators and cytokines enter the bloodstream, induce a short-lived systemic inflammatory response syndrome and lead to SAEs. This explanation assumes the same mechanisms as COVID-19 related syndromes [183]. However, this could not explain why thrombosis is more frequent in adenoviral vaccines and not in other vaccines. Another group of researchers claimed that vector-based vaccines could generate alternatively spliced spike proteins that are secreted to the systemic circulation, where they cause thrombosis. This explanation is called vaccine-induced COVID-19 mimicry syndrome [192]. However, spike proteins are also found in the sera of recipients of non-vector-based vaccines such as mRNA-1273 [193]. While the above studies offer possible explanations, they lack critical and direct evidence.

Recent studies suggest a potential mechanism of VITT reminiscent of heparin-induced thrombocytopenia (HIT). HIT is a complication of heparin treatment that usually occurs 5 to 10 days after heparin exposure. It is characterized by venous thromboembolism and thrombocytopenia. After heparin exposure, the negatively charged heparin binds to the positively charged platelet factor 4 (PF4), forming an epitope and resulting in the production of anti-PF4 antibodies. Anti-PF4 antibodies form an immunocomplex with PF4 and bind to the Fc receptor of platelets, in turn activating the platelets. The activated platelets release more procoagulant proteins and cytokines including PF4. Uncontrolled platelet activation and aggregation lead to thrombosis and subsequent platelet clearance from the circulation leads to thrombocytopenia [194].

Similar to HIT, VITT is associated with IgG antibodies against PF4 [189, 195]. However, the anti-PF4 antibodies in VITT target a different epitope from those in HIT [196]. VITT usually occurs later than HIT, ranging from 5 to 30 days (commonly 10 to 16 days) following vaccination [197]. It is not clear how anti-PF4 antibodies are induced in VITT, but researchers have proposed that the adenoviral vector in the vaccine plays a role. Same as heparin, the viral coat of the adenoviral vector is negatively charged and was found to form stable complexes with PF4 [198, 199]. In addition, Baker et al. found a positive relationship between the negative charge on the viral coat and the frequency of thrombosis [198].

**Fig. 4:**
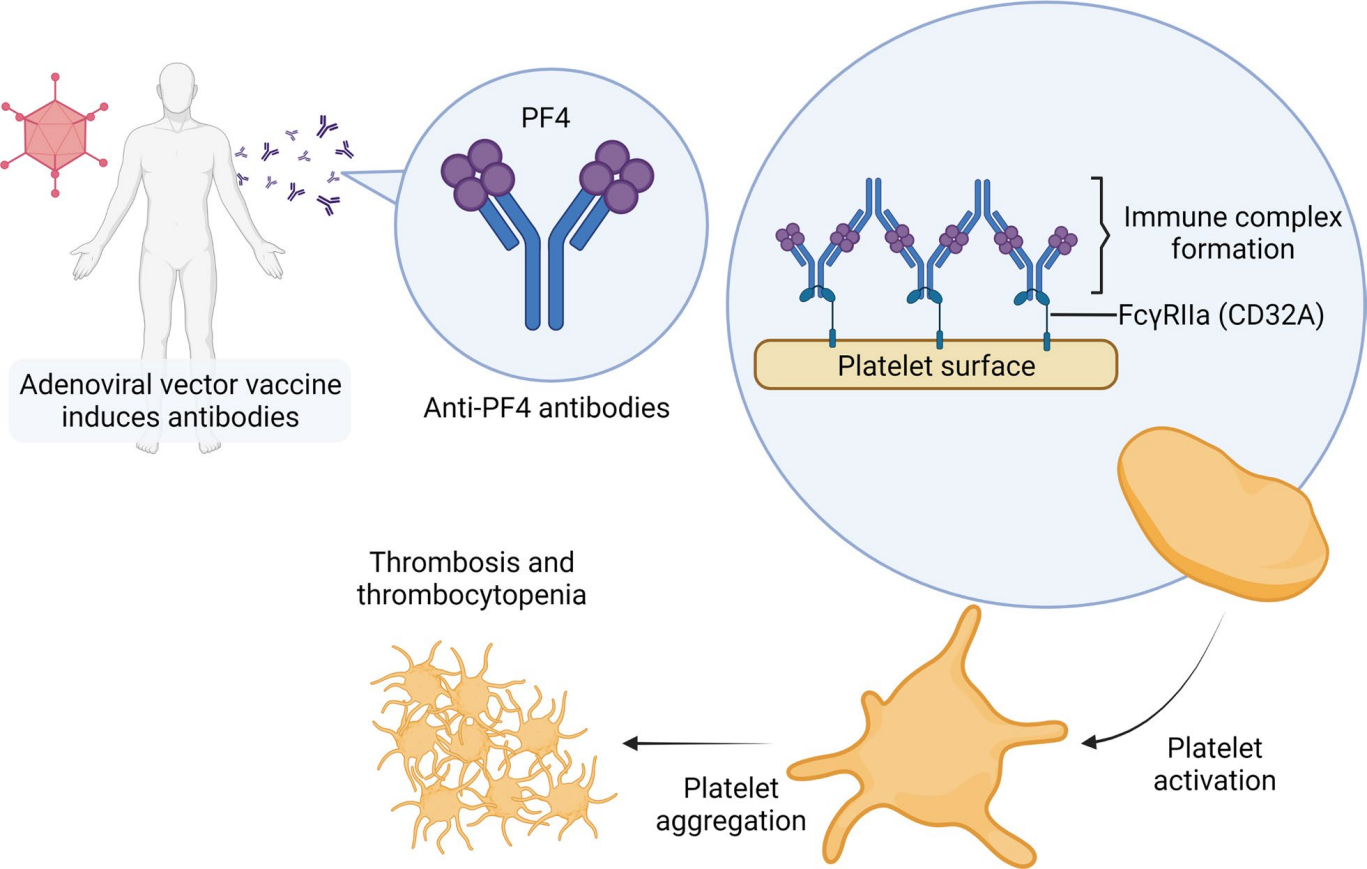
Potential mechanism of VITT. After vaccination, the viral proteins in the vaccine components might bind with PF4, thereby forming epitopes that can be recognized by the immune system and inducing the generation of anti-PF4 antibodies. Anti-PF4 antibodies form immune complexes with PF4 and bind with the Fc receptor on the surface of the platelet, leading to platelet activation and aggregation, and finally VITT 5 to 20 days after vaccination. (Created with BioRender.com)

Taken together, this evidence suggests that the mechanism of VITT might include two steps (Fig. 4). In the first step, the viral proteins in the vaccine components form complexes with PF4 and form epitopes that can be recognized by the immune system. Consequently, anti-PF4 antibodies are induced, in turn forming immune complexes with PF4 and binding with the Fc receptor on the surface of the platelet, leading to platelet activation, aggregation, and VITT 5 to 20 days after vaccination [199].

### Myocarditis and pericarditis in mRNA vaccines

mRNA vaccines such as Pfizer’s BNT162b2 and Moderna’s mRNA-1273 have been reported to be associated with a higher risk of myocarditis or pericarditis [200–203]. The excessive risk was about 2.7 (1 to 4.6) events per 100,000 persons [203]. In contrast, SARS-CoV-2 infection leads to about 11 (5.6 to 15.8) myocarditis events per 100,000 persons, posing a much higher risk of myocarditis in addition to the risk for additional SAEs [203]. Cumulative reports have concluded that myocarditis following mRNA COVID-19 vaccination usually happens within a week of receiving the vaccination, more often after the second dose. The occurrence of myocarditis is more frequent in male adolescents and young adults, and is usually self-limiting and uneventful [200, 202, 204, 205]. Potential mechanisms of post-vaccination myocarditis or pericarditis include hormonal differences, mRNA immune reactivity, and antibody cross-reaction with myocardial proteins [206] although the exact mechanism remains elusive.

### Adverse events in children

Currently, the US FDA has authorized mRNA vaccines in children over 6 months of age. One concern of mRNA vaccines is the risk of myocarditis and pericarditis, which are known to be more frequent in young male recipients in the subgroup aged 12–17 years old [207, 208]. So far, no severe adverse event has been reported in randomized controlled trials in children and adolescents [15, 21, 22, 209]. However, these clinical trials contain a smaller number of participants and thus may not be able to identify rare severe adverse events. Hause et al. analyzed the US safety monitoring systems and identified low incidence of myocarditis (2.2 per million doses) in children aged 5 to 11 years during the first 4 months after vaccination, which is about 10 times lower than adolescents and adults [210]. In most cases of reported vaccination-associated myocarditis in children are mild with quick recoveries [211].

### Adverse events related to booster shots

Since the administration of boosters, there have been discussions and concerns about whether or not there will be additional adverse events or increased side effects after homologous or heterologous boosters. For example, whether or not a booster of the adenoviral vector vaccine or mRNA vaccine exposes the recipient to additional risks of VITT or myocarditis, respectively. A review of ChAdOx1 vaccine recipients showed that the rate of VITT within 14 days of the first dose was 8.1 per million doses, while that of the second dose was 2.3 per million doses [212], suggesting that VITT is an idiosyncratic condition and the second dose or the booster might be safe in people who did not develop VITT after the first dose. Similarly, myocarditis was rarely reported following an mRNA vaccine booster dose [213]. As for SAEs after heterologous boosters, there is limited evidence to conclude whether or not there are additional risks of VITT or myocarditis, but people with higher risks of thrombosis or myocarditis were suggested to avoid adenoviral vector or mRNA vaccines as their booster dose [214, 215].

Solicited adverse events from the boosters are similar to that of the first two doses. CDC has reported that local and systemic adverse events were less frequent in people receiving a homologous mRNA vaccine booster than in people who received a heterologous booster [213]. While some studies showed that the adverse events were similar across heterologous or homologous booster groups [167], other studies showed a greater adverse effect and systemic reactogenicity after a heterologous second prime or booster dose than a homologous one [156, 165]. Thus, the results are inconsistent and more data might be needed before we reach a solid conclusion. Note that many of the systemic adverse events are self-reported and could be related to the nocebo effect, as pointed out in a study [216].

Overall, studies have suggested that homologous and heterologous boosters are safe, but the long-term data on safety are still limited and further surveillance is needed.

## Immune correlates of protection (Immunobridging)

Another important COVID-19 vaccine topic would be to apply the concept of immune correlates of protection to the development of COVID-19 vaccines. The idea of an immune correlate of protection (or immuno-bridging) is to use immunological measurements as a predictive marker for the protective effect against infectious diseases [217]. One major barrier to the development of the new COVID-19 vaccine is the expensive and long process of running large-cohort phase III efficacy studies [218]. In addition, as the first COVID-19 vaccines become available on the market, it becomes an ethical dilemma for the late-coming COVID-19 vaccine developers to conduct randomized controlled efficacy trials [218]. Applying correlates of protection can potentially solve these problems and greatly facilitate the development of COVID-19 vaccines. Moreover, from our current understanding, the prevention of infection is mostly driven by neutralizing antibodies, but the prevention of severe diseases is likely to involve a complex functional profile of SARS-CoV-2 binding antibodies and also different kinds of SARS-CoV-2 targeting T and B cells [219]. Studying immune correlates of protection can help us better understand the mechanism of action of vaccine-induced protection for preventing both infection and severe diseases, which can be used to guide future vaccine development and immunotherapies for COVID-19.

As of August 2022, there have only been limited immune correlation studies for SARS-CoV-2. One large-scale health worker study has shown that antibody positivity is a good predictor of lower infection risk, suggesting that antibody level by itself can be used as a protective marker [220]. However, the authors of this study did not make antibody level comparisons between infected and non-infected people, so we cannot deduce quantitative antibody information from this study. Another two studies conducted by Khoury et al. and Earle et al*.* have compared 7 COVID-19 vaccines under EUA and found that the vaccine-induced neutralizing antibodies have a strong correlation with the protective effect in their phase III clinical trial [221, 222]. However, there are two caveats in both studies worth mentioning. One is that the antibody measuring methods for different vaccines are not standardized, so the authors had to use convalescent antibody levels measured in each study to normalize the vaccine-induced antibody, which is not an ideal way for quantification. The other caveat is that the clinical trials for these seven COVID-19 vaccines are conducted in different countries at different times and therefore the vaccinated population and the prevalent viral strains are not the same across these studies, which could potentially confound the efficacy results. Finally, both Moderna and AstraZeneca groups have published their immune correlates of protection studies by following up on their vaccine recipients [223, 224]. They both found that the spike protein-binding antibodies and neutralizing antibodies induced by mRNA-1273 and ChAdOx1 vaccines are good predictors for symptomatic COVID-19 protection. Furthermore, their data provide absolute antibody levels that can be used to predict vaccine efficacy, which can be applied to guide vaccine regimen modifications and the frequency of booster vaccination. However, the limitations of both studies are the generalizability of their correlations of protection. Since both the Moderna and AstraZeneca groups do not include different kinds of vaccines in their correlation studies, the antibody threshold levels found in their studies might not apply to other vaccines. In addition, as new SARS-CoV-2 strains continue to appear, it is expected that the neutralizing antibody—protective efficacy relationship would change in these mutant strains, and updated correlates of protection studies would need to be performed.

There are many methods the vaccine industry and governments can collaborate on to better understand correlates of protection from COVID-19 infection [225]. First, standardizing the methods of antibody measurement in different vaccine studies could greatly facilitate cross-trial data collection of correlates of protection. The WHO could play a leading role in defining standard antibody measurement methods. In addition, although the prevention of symptomatic COVID-19 is mainly driven by neutralizing antibodies, the prevention of severe COVID-19 diseases is likely to be contributed by multiple immune components, including CD4+ T cells, CD8+ T cells, and memory B cells [219, 226–228]. For example, neutralizing antibodies wane over time but cellular immunity and memory B cells likely persist and provide long-term surveillance [226, 229–231]. Simultaneous measurement of multiple immune markers in vaccine trials would allow us to conduct correlates of protection studies on both symptomatic and severe disease prevention in a more systematic way. Moreover, the generalizability issue is the major limitation that prevents the industry from applying correlates of protection findings to the development of new vaccines. To expand the generalizability of correlates of protection findings, it is important to coordinate immune correlate studies that include participants receiving different types of COVID-19 vaccines.

In summary, finding surrogate immunological markers for measuring the efficacy of the COVID-19 vaccine is likely to be the future trend, as most mature vaccines, such as the influenza vaccine or HPV vaccine, are not required to repetitively perform randomized controlled efficacy studies for their new vaccines [217]. Currently, a few studies have suggested that neutralizing antibodies is a good predictor of protection efficacy, and the Moderna and AstraZeneca studies have provided absolute antibody levels that can be used to predict protective efficacy for people receiving the same vaccine [220–224]. The next step for this field would be to perform immune correlation studies that include different vaccines using a similar immunogenic mechanism (e.g. correlation studies include multiple mRNA vaccines), which will become valuable evidence to guide future development of vaccines of the same kind. We hope that more cross-vaccine immuno-bridging data will become available soon to accelerate the development of the next generation of COVID-19 vaccines.

## Next-generation vaccine

The current COVID-19 vaccines have been proven highly effective in curbing the pandemic; however, emerging variants are becoming more transmissible and are better at escaping from vaccine-induced immunity. Although booster vaccination offers improved protection, repeated boosting with current vaccines based on the ancestral SARS-CoV-2 virus is unsustainable. An Israeli study suggested that the immunogenicity of mRNA vaccines peaks after three doses and that the fourth dose of mRNA vaccines only offers marginal protection in young adults [232]. In line with this, a fourth dose of BNT162b2 showed short-term and modest protection against Omicron infection in a real-world assessment, although the protection against hospitalization did improve relative to three doses [150, 151]. Therefore, new vaccines are being developed in the hope of outpacing viral evolution. In this section, we discuss recent next-generation vaccine candidates that are undergoing clinical development.

### SARS-CoV-2 variant-specific booster

The need for a variant-specific vaccine was first raised when the Beta variant was found to moderately escape neutralization by monoclonal antibodies as well as convalescent and vaccinated sera [233]. Although available COVID-19 vaccines were later shown to be effective against Beta [234], several new SARS-CoV-2 variants have been reported to partially escape natural or vaccine-induced immunity. The effectiveness of COVID-19 vaccines against symptomatic infection of Delta and Omicron variants is notably reduced, though remain highly effective in preventing severe disease and hospitalization [76, 77, 94, 235]. Owing to the flexibility of RNA technology, Moderna has been testing several variant-specific and bivalent booster vaccines, including mRNA-1273.351 (Beta), mRNA-1273.617 (Delta), mRNA-1273.529 (Omicron), mRNA-1273.211 (original + Beta), mRNA-1273.213 (Beta + Delta), and mRNA-1273.214 (original + Omicron) (NCT04927065). Pfizer and BioNTech also developed Beta-, Delta-, and Omicron-specific vaccines [236]. Both companies initiated clinical trials of their Omicron-specific vaccines in late January 2022. AstraZeneca also tested a Beta-specific version of its vaccine, called AZD2816, in a phase II/III trial starting in June 2021 (NCT04973449) [237], but it was terminated in February 2022 when Omicron became the prominent strain. Finally, Johnson & Johnson developed Beta- and Omicron-based vaccine candidates, named Ad26.COV2.S.351 and Ad26.COV2.S.529, respectively. There are limited published clinical results of these variant-specific boosters. Moderna reported that boosting with their original or Beta-specific vaccine both increased the nAb titers compared to 1 month after the primary series [238].

Omicron-specific vaccines have been tested in animals while human trials are ongoing. Some small-scale animal studies found Omicron-specific mRNA-based boosters offer little advantage over the original vaccines in rhesus macaques and mice that were previously immunized with 2 doses of the original vaccine [239, 240]. Nonetheless, boosting with Omicron-specific mRNA (mRNA-1273.529) and Ad26 viral vector (Ad26.COV2.S.529) vaccine candidates both induced stronger Omicron-neutralizing responses in pre-immunized rodents when compared to boosting with the original vaccine [240, 241]. Additionally, the timing of boosting should be optimized, as preexisting immunity may compromise the effect of the booster dose [130, 242]. Notably, animal studies also found Omicron-specific vaccines induced potent antibodies and immunity against Omicron but not the ancestral strain in vaccine-naive mice and Syrian hamsters [241–243]. A serological study of vaccine-naive individuals infected with Omicron also showed that their sera contain nAbs against Omicron but not the other variants [244]. These studies indicated that Omicron is a highly divergent variant with strong immune evasion and little cross-reactivity with the earlier variants. Therefore, unlike the previous variants, an Omicron-specific vaccine may be particularly needed.

Some positive data on Omicron-specific boosters have been reported by the pharmaceutical companies. Moderna’s bivalent mRNA-1273.214 (WT + Omicron) induced higher nAb titers against Omicron and BA.4/5 [245–247]. Among seronegative participants 1 month after boosting, mRNA-1273.214 induce 1.6-fold higher neutralizing GMT against Omicron than mRNA-1273 (2372 [CI: 2071, 2718] vs. 1473 [CI: 1271, 1708]). While the GMT against BA.4/5 was threefold lower than that against BA.1, Pfizer-BioNTech’s Omicron-adapted vaccine candidates also induced higher nAb responses against Omicron [248]. A monovalent Omicron-adapted candidate tested at 30 µg and 60 µg doses induced 2.2- and 3.2-fold higher nAb GMT against Omicron than BNT162b2, respectively. A bivalent candidate at 30 µg and 60 µg induced 1.6- and 2.0-fold higher nAb GMT against Omicron, respectively. However, these Omicron-specific vaccine candidates induce fewer neutralizing effects against newer SARS-CoV-2 variants. The Moderna mRNA-1273.214 induced threefold lower neutralizing GMT against BA.4/5 than that against BA.1. Similarly, Pfizer also found that sera from participants boosted with its Omicron-adapted vaccine candidates neutralized BA.4/5 threefold less efficiently than BA.1 in live virus neutralizing assays. These discoveries are consistent with reports showing that BA.4/5 escape neutralizing antibodies induced by Omicron BA.1 infection [124].

In a possible future where COVID-19 becomes endemic, we may adapt the influenza vaccination strategy, where people receive periodic boosters against the dominant circulating variant to ensure effective immune protection.

### Pan-coronavirus vaccine

The rapid emergence of variants adds uncertainty to the development of virus-specific vaccines, as a new variant may upend the efforts made on the previous one. Therefore, pan-coronavirus vaccines are being developed to offer protection against SARS-CoV-2 variants and coronaviruses that may emerge in the future. That serum samples from people who had been infected with SARS-CoV in the 2003 endemic showed neutralizing activity against the SARS-CoV-2 supports the feasibility of a universal vaccine against betacoronaviruses [233, 249, 250]. Several approaches have been proposed or employed for the development.

Nanoparticle formulations have been developed to induce more potent immunogenicity by presenting antigens in arrays on the surface of nanoparticles that resemble the conformation of real virus particles. Owing to the strong immunogenicity some nanoparticle vaccines were shown to induce cross-reacting nAb. For example, the Spike Ferritin Nanoparticle (SpFN) COVID-19 vaccine created by researchers at the Walter Reed Army Institute of Research consists of a fusion protein of *Helicobacter pylori* ferritin linked to the C-terminus of pre-fusion stabilized ectodomain (residues 12–1158) of the SARS-CoV-2 spike protein that self-assembles into nanoparticles. In a preclinical study, the SpFN vaccine induced nAb against SARS-CoV-2, variants of concern, and SARS-CoV-1 [251]. The vaccine is currently in phase I clinical trial (NCT04784767). Since cross-reacting nAbs have been reported to target the spike RBD [252, 253], self-assembling nanoparticles were also used to elicit potent RBD-specific cross-nAbs [254–256].

Cross-reacting antibodies may also be induced by combining antigens of coronaviruses from different clades. This strategy is supported by a report that potent pan-sarbecovirus nAbs were found in convalescent SARS-CoV-1 patients who have received BNT162b2 [257]. Further research is required to show whether priming with a SARS-CoV-2 vaccination followed by a SARS-CoV-1 booster will produce a similar pan-coronavirus immune response. This cross-clade strategy can be combined with the aforementioned nanoparticle technologies. For example, Cohen et al. developed multivalent nanoparticles co-displaying RBDs of up to 8 different coronaviruses, dubbed mosaic-8. The multivalent vaccine induced nAbs against zoonotic coronaviruses that were not displayed on the nanoparticles, suggesting potential protection against emergent coronaviruses that may potentially spill over to humans [258]. In an animal study, mosaic-8 immunization elicited neutralizing antibodies against SARS-CoV-2 variants, including Omicron to a similar degree, and provided protection from SARS-CoV-2 challenges [259]. Moreover, pan-coronavirus nAbs can also be induced by chimeric spike proteins. Martinez et al. fused RBD, NTD, and S2 domains from different coronaviruses into bi- or tri-valent immunogens to be encoded by an mRNA vaccine. Although the chimeric spike mRNA vaccine induced lower nAbs against SARS-CoV-2, it nonetheless provided better immune responses against multiple sarbecovirus clades than monovalent SARS-CoV-2 in mice [260]. Thus, these preclinical studies have demonstrated the feasibility of using multivalent coronavirus vaccines to provide comprehensive protection against emergent zoonotic coronaviruses.

Another strategy is to target conserved regions of coronavirus. The conserved regions are typically buried in the three-dimensional protein structure or encapsulated inside the viral particle, making them hard for antibodies to bind and neutralize. Nonetheless, immunizing with these antigens can induce T cell immunity and provide an extra layer of protection [261, 262]. Preexisting ORF1- and N (nucleocaspid)-specific memory T cells primed by endemic human coronaviruses (hCoVs) were shown to cross-react with and protect against SARS-CoV-2 infection [263]. Long-lasting T cell immunity in convalescent SARS-CoV-1 patients also displayed cross-reactivity to N protein of SARS-CoV-2 [264]. Therefore, vaccines targeting non-spike antigens could be another way leading to pan-coronavirus protection. Examples of vaccines undergoing clinical development include candidates being developed by Gritstone and ImmunityBio, both delivering nucleocaspid in addition to spike antigen to stimulate T cell immunity. In addition to N protein, other potential targets were also identified using reverse vaccinology (RV), which predicts immunogenic epitopes from the genome of the pathogens [265]. For instance, the Vaxign-ML RV model predicted non-structural proteins (NSPs) Nsp3 and Nsp8, in addition to S protein, to induce high immunity [266]. Thus, non-structural protein antigens may also be immunized along with S protein to provide a more comprehensive protection. Similarly, these additional antigen targets can be used to generate multivalent vaccines, multiepitope peptide vaccines, or mosaic nanoparticle vaccines [267].

## Conclusions

More than two years have passed since SARS-CoV-2 emerged and became the biggest pandemic in the twenty-first century. The first case happened in December 2019 and soon after that, human-to-human transmission was confirmed. However, the WHO was reluctant to announce a pandemic until March 2020 [268]. The WHO and US CDC were also late to announce mask mandates although it was known that COVID-19 could spread via airborne droplets. Despite the initial delayed public health responses, the scientific, medical, and biopharmaceutical communities were able to collaborate to develop vaccines, diagnostics, and therapeutics. The scientific community has shown the importance of a more open and collaborative environment for sharing scientific findings in the form of preprints or social media. Many biopharmaceutical companies also did not enforce patents to ensure more affordable and widely-distributed vaccines. Massive vaccination efforts and physical distancing helped to constrain the spread of COVID-19 and ease the severity and transmission of the disease.

Although Omicron seems to cause milder symptoms than previous variants in people who are fully vaccinated and boosted, we do not know if there will be yet another variant with more severe symptoms and the same degree of immune escape. It is also possible that SARS-CoV-2 will become endemic and continue to circulate within the population in the foreseeable future. Therefore, variant-specific or pancoronavirus vaccines and future boosters might be required due to waning immunity and breakthrough infections from new variants. To develop next generation vaccines, it is important to establish immune correlates of protection using nAb titers and additional biomarkers such as cellular immunity or memory B cell levels. As more and more people are vaccinated and countries start to lift their mask mandate, ensuring vaccine equity around the globe, especially in the low- and middle-income countries, will become increasingly important.

This COVID-19 pandemic has changed the way we live, and it will continue to shape our future life. People are conceptually more willing to be vaccinated and wear masks in daily life and remote working and learning will probably become a feasible option in the future. Whether or not we will encounter another pandemic this century is unknown but, just in case, we will need to be prepared mentally, scientifically, and infrastructurally.

## Supplementary Information

**Additional file 1: Table S1.** Vaccine effectiveness against viral variants. Studies of vaccine effectiveness against viral variants. Related to Table 2.

**Additional file 2: Table S2.** Vaccine efficacy and effectiveness in children and adolescents

**Additional file 3: Table S3.** Vaccine booster effectiveness against viral variants. Studies of vaccine booster effectiveness against viral variants. Related to Table 2.

**Additional file 4: Table S4.** Heterologous booster lab data. Studies of heterologous vaccine boosters and their immunogenicity. Related to Table 3.

## Data Availability

Not applicable.

## Abbreviations

COVID-19: Coronavirus Disease 2019
SARS-CoV-2: Severe Acute Respiratory Syndrome Coronavirus 2
EUA: Emergency Use Authorization
EUL: Emergency Use Listing
WHO: World Health Organization
m1Ψ: N1-methylpseudouridine
GMT: Geometric mean titer
tPA: Tissue plasminogen activator
alum: Aluminum hydroxide
nAb: Neutralizing antibody
VoC: Variants of concern
ACE2: Angiotensin-converting enzyme 2
RBD: Receptor-binding domain
NTD: N-terminal domain
CDC: Centers for Disease Control and Prevention
SAE: Severe adverse event
VAERS: Vaccine Adverse Event Reporting System
AEFI: Adverse event following immunization
AESI: Adverse event of special interest
VITT: Vaccine-induced immune thrombotic thrombocytopenia
HIT: Heparin-induced thrombocytopenia
PF4: Platelet factor 4
SpFN: Spike Ferritin Nanoparticle
hCoVs: Human coronaviruses
MIS: Multisystem inflammatory syndrome
ICU: Intensive Care Unit
RV: Reverse vaccinology
mRNA: Messenger
CDC: Centers for Disease Control and Prevention
FDA: Food and Drug Administration
